# Letter on ‘linear accelerator-based stereotactic arrhythmia radioablation for paroxysmal atrial fibrillation in elderly: a prospective phase II trial’

**DOI:** 10.1093/europace/euae138

**Published:** 2024-05-31

**Authors:** Oliver Blanck, Marcin Miszczyk, Pieter G Postema

**Affiliations:** Department of Radiation Oncology, University Medical Center Schleswig-Holstein, Arnold-Heller-Strasse 3, Kiel 24105, Germany; Department of Urology, Comprehensive Cancer Center, Medical University of Vienna, Spitalgasse 23, Wien 1090, Austria; Collegium Medicum—Faculty of Medicine, WSB University, Zygmunta Cieplaka 1c, Dąbrowa Górnicza 41-300, Poland; Department of Cardiology, Amsterdam UMC, University of Amsterdam, Meibergdreef 15, Amsterdam 1105AZ, The Netherlands

With great interest, we read the results of the prospective clinical trial investigating StereoTactic Arrhythmia Radioablation (STAR) for the treatment of Atrial Fibrillation (AF) by Di Monaco and *et al*.^1^ from Bari, Italy. STAR offers a potential novel approach to refractory arrhythmias as exemplified in selective cases and small series of patients with cardiomyopathy and recurrent malignant ventricular tachycardia (VT) despite unsuccessful treatment with antiarrhythmic drugs and (often multiple) catheter ablations.^2,3^ Importantly, STAR for VT is still considered experimental^4^ and should preferably be performed in clinical trials and registries, as e.g. initiated in the STOPSTORM.eu consortium.^5^

Although we are very excited by the potential of STAR, the study by the Italian colleagues evaluated STAR for the treatment of a ‘benign’ arrhythmia: AF. However, in our view, STAR should currently be restrained to life-threatening conditions, that appear otherwise extremely difficult to manage. While AF can heavily impact on quality of life, and at older age often associates with other cardiac and extra-cardiac morbidities, there are multiple therapies available to reduce AF-burden and AF-related symptoms. These include antiarrhythmic drugs, catheter ablation, and pace-and-ablate strategies, all of which can also be considered in older patients. Therefore, we find it concerning that, as opposed to VT-STAR trials, the authors excluded patients previously treated with ablation.

Importantly, even if STAR had no potential downsides, we would still worry about its use in AF as the biological mechanisms of STAR are not fully understood.^6^ When fibrous lesions are the goal, even higher doses and better precision of radiation delivery are likely necessary.^7,8^ Moreover, in a condition without (severely) reduced life expectancies like AF, potential mid- to long-term STAR side-effects to surrounding critical structures are extremely important. The adjacent gastro-intestinal tract is radiation-sensitive, and VT-STAR already resulted in a radiation-related death at six months.^9^ Moreover, most of the patients included in this trial had valve dysfunctions, while these could well worsen even beyond one year after STAR.^10^ Thus, we wonder why a 1-month period was chosen to evaluate safety, as opposed to VT-STAR trials dealing with higher morbidity and mortality. Of note, the authors report that ‘at a median follow-up of 16 months, no acute toxicity more than Grade 3 was reported’. However, later in the manuscript we find that there were three Grade 4 events, including a possibly treatment-related Grade 4 event 1 h after STAR. Finally, in STAR for VT currently no comparable cohorts are available, as the treatment is regarded as bail-out. In this trial, the results could have been compared to, e.g. the remaining non-STAR-treated patients.

In conclusion, although we are actively using STAR for selected cases with malignant cardiac disease, we raise concerns about the use of STAR for a ‘benign’ condition like AF with multiple other treatment options, especially in the setting of available standard-of-care treatment methods. We thus strongly urge the community to delay further efforts of STAR for AF until long-term efficacy and safety of the trial by Di Monaco and colleagues are presented in full detail.
